# Evaluation of serum amino acids and non-enzymatic antioxidants in drug-naïve first-episode major depressive disorder

**DOI:** 10.1186/s12888-020-02738-2

**Published:** 2020-06-24

**Authors:** Md. Rabiul Islam, Samia Ali, James Regun Karmoker, Mohammad Fahim Kadir, Maizbha Uddin Ahmed, Zabun Nahar, Sardar Mohammad Ashraful Islam, Mohammad Safiqul Islam, Abul Hasnat, Md. Saiful Islam

**Affiliations:** 1 Department of Clinical Pharmacy and Pharmacology, Faculty of Pharmacy, University of Dhaka, Dhaka, 1000 Bangladesh; 2 Department of Pharmacy, University of Asia Pacific, 74/A Green Road, Farmgate, Dhaka, 1205 Bangladesh; 3 Department of Pharmacy, Noakhali Science and Technology University, Sonapur Noakhali, 3814 Bangladesh

**Keywords:** Major depressive disorder, MDD, Serum, Amino acids, Non-enzymatic antioxidants

## Abstract

**Background:**

The alterations of biological markers are thought to be effective tools to understand the pathophysiology and management of major depressive disorder (MDD). A lot of researches has implied many markers for depression, but any of them fully discovered the association between the markers and depression. The present study investigated the serum levels of amino acids and non-enzymatic antioxidants in major depression, and also explained their association with depression.

**Methods:**

This study examined 247 MDD patients and 248 healthy controls (HCs) matched by age and sex. The Hamilton Depression Rating Scale (Ham-D) was used to all the participants to measure the severity of depression. Quantification of serum amino acids, vitamin A and E were carried out using the HPLC system whereas vitamin C levels were measured by UV-spectrophotometer. All the statistical analysis was performed by SPSS statistical software (version 23.0). The independent sample t-test, the Mann-Whitney U test, and the Fisher’s exact test were applied to detect the group differences where a Bonferroni correction applied to the *p* value.

**Results:**

It was observed that serum levels of four amino acids (methionine, phenylalanine, tryptophan, and tyrosine) along with three non-enzymatic antioxidants (vitamin A, E, and C) were significantly dropped in MDD patients compared to HCs (Cohen’s d (d): − 0.45, − 0.50, − 0.68, − 0.21, − 0.27, − 0.65, and − 0.24, respectively). Furthermore, Ham-D scores of cases were negatively correlated with serum levels of methionine (*r* = − 0.155, *p* = 0.015) and tyrosine (*r* = − 0.172, *p* = 0.007).

**Conclusion:**

The present study suggests that lowered serum methionine, phenylalanine, tryptophan, tyrosine, and non-enzymatic antioxidants are associated with depression. The reduction of these parameters in MDD patients may be the consequence, and not the cause, of major depression.

## Background

Major depressive disorder (MDD) is a tremendously growing mental health issue with a prevalence of 5.5% in women and 3.2% in men worldwide [1]. In Bangladesh, WHO reported 4.1% of the adult population has depression [2]. Several factors, such as genetics, alterations in brain structure and function, changes in biological markers, and nutritional status contribute to the development of MDD or increase the risk of developing the disorder [3–6]. Even though many propitious biological mechanisms have been proposed, as the monoamine hypothesis, hypothalamic-pituitary-adrenal (HPA) axis deregulation, and chronic inflammation, the pathophysiology of MDD remains ambiguous; and there are no established biomarkers [7].

Variations in serotonergic, noradrenergic, dopaminergic, glutamatergic, and g-aminobutyric acid (GABAergic) systems are observed in psychiatric disorders [8–10]. These neurotransmitters are either amino acids or are synthesized from amino acids. It is well known that the neurotransmitters serotonin and norepinephrine are intimately related to depression. The precursors of these neurotransmitters are the amino acids tryptophan and tyrosine, respectively and depleted levels of these amino acids are observed in MDD patients [11]. Amino acid metabolism has the potential for understanding the pathogenesis and predicting the therapeutic response in MDD [12]. Amino acid supplements usually reduce or alleviate the symptoms of depression and other mental illnesses as they can convert into neurotransmitters [13]. The amino acids tryptophan, tyrosine, and phenylalanine are thought to play a vital role in the pathogenesis of depression [14]. Methionine helps the body to produce S-adenosyl-l-methionine (SAMe) which is found to be lower in depressed patients according to two studies while another study suggests that the depletion was not so significant [15–17].

Over the recent years, free radical chemistry has got a myriad of attention because a balance between the production of free radicals by several endogenous mechanisms and antioxidant activities is a prerequisite for maintaining proper physiological function [18]. The oxidative stress shows an important role in depression and the antidepressant activity is mediated by the antioxidant function [19]. Generally, antioxidants impede the process of lipid peroxidation by deactivating free radicals. Antioxidants are categorized into non-enzymatic antioxidants and enzymatic antioxidants [20]. The primary defense is provided by enzymatic antioxidants while the secondary defense is by non-enzymatic antioxidants like vitamins A, E, and C [21]. Any deficiency of these antioxidants mainly non-enzymatic antioxidants are considered to be the causative factors for oxidative stress and in the long run, it is manifested in the development of MDD [22–24]. Patients with depressive disorders have an imbalance between the production and neutralization of reactive oxygen species (ROS) [25]. Depleted levels of antioxidants mainly vitamin A, E, and C appear to be the possible cause of depressive disorder [26]. Moreover, a meta-analysis reported that MDD patients had lower levels of the antioxidant uric acid than controls [27]. Nevertheless, the results are contrary. A study showed there was no difference between the patients and healthy volunteers concerning plasma levels of vitamin A, E, and C [28]. Whereas another study showed that levels of vitamin E in the serum of MDD patients were lower as compared to healthy volunteers [29].

The clinical relevance of the above assessment regarding the association of serum levels of amino acids and non-enzymatic antioxidants with MDD is still obscure. The present study aimed to observe the association of serum levels of these parameters in major depression.

## Methods

### Participants

This is a prospective case-control study conducted in the department of psychiatry, Bangabandhu Sheikh Mujib Medical University (BSMMU), Dhaka, Bangladesh. MDD patients participated from both psychiatric outpatients (57%) and inpatients (43%) of BSMMU. Only drug-naïve first-episode MDD patients who met the diagnostic criteria were included in the current study. Healthy controls (HCs) were recruited from the different areas of Dhaka city matched by age, sex, and body mass index (BMI). Based on recruitment probabilities, subjects were individually randomized to be recruited in this study. Diagnosis of MDD patients and evaluation of HCs were performed by a qualified psychiatrist based on the Diagnostic and Statistical Manual of mental disorders, 5th edition (DSM-5). The exclusion criteria applied to all the study participants were addiction, infectious disease, excessive obesity, mental retardation or psychiatric illness, liver, or kidney failure. Non-cooperative MDD patients who denied sharing their clinical data and blood samples were also excluded from this study. Additionally, we ensured the study participants were free from taking any medication that could interfere with the serum levels of the amino acids and anti-oxidant vitamins. Socio-demographic and biographical profiles of the study population were obtained based on pre-designed questionnaires. The Hamilton Depression Rating Scale (Ham-D) was applied to all the cases to measure the severity of depression.

The protocol was approved by the ethical review committee and the investigations were conducted according to the principles expressed in the Declaration of Helsinki. The participants were well informed about the aim of the study and deferred written consent was obtained from all the patients or caregivers and healthy volunteers.

### Blood sample collection

Blood samples (5 ml) were drawn from the cephalic vein of each participant after an overnight fast. The samples were allowed to clot for an hour at room temperature. It was centrifuged at 1000 g for 15 min at room temperature, serum was then extracted from the supernatant, placed into microtubes, and stored at − 80 °C until analysis.

### Chemicals and reagents

All the chemicals and reagents used were of analytical grade, obtained from commercially available companies. Standards of amino acids and antioxidant vitamins were purchased from Sigma-Aldrich, Inc. The other supportive chemicals of the recommended grade were provided by the clinical pharmacy and pharmacology department, University of Dhaka, Bangladesh.

### Quantification of amino acids and non-enzymatic antioxidants

Serum levels of amino acids were measured by high-performance liquid chromatography (HPLC) system (Dionex Ultimate 3000, Thermo Fisher Scientific Inc., USA) with UV detection. Briefly, an aliquot of 40 μL serum sample was mixed with 10 μL H_2_SO4 in a tube. Then vortexed for the 30 s at room temperature, and then centrifuged at 10000 g for 2 min at 4 °C for protein precipitation. Ten microliter supernatant was transferred into a clean tube and mixed with a 40 μL borate buffer (5 mmol/L, pH 8.5) then vortexed and centrifuged with the same specification. Five microliter iTRAQ reagent (AB SciexPte. Ltd) was added with the mixture and vortexed and centrifuged accordingly. The tubes were then incubated for at least 30 min at room temperature. Then the 5 μL NH_2_OH and at last 30 μL internal standard were added to each tube then vortexed and centrifuged in the same way. For derivatization, 5 μL of the supernatant was injected carefully into the chromatography on a C_18_ column with the mobile phase consisted of water including 0.1% formic acid (solvent A) and acetonitrile including 0.1% formic acid (solvent B). The flow rate was 0.8 mL/min. The total runtime was 15 min. We transformed the spectral map into the content of each amino acid and saved it in an excel file format. And then it was used for pattern recognition [30].

Vitamin A and vitamin E were determined from serum samples by liquid-liquid extraction using n-hexane and evaporated to dryness using a sample concentrator (DB-3, Techne, UK) at 40 °C under a stream of nitrogen. Then, the dried extract was reconstituted in the mobile phase. The serum concentrations of retinol and α-tocopherol were measured simultaneously at 291 nm by the previously described modified RP-HPLC method with UV detection [31]. From the reconstituted sample, 20 μL was injected carefully into the chromatography on a C_18_ column with acetonitrile: methanol (75:25) mobile phase flowing at 1 ml/min. To analyze serum vitamin-C, extracted serum was treated properly with 5% trichloroacetic acid (TCA) in a test tube and centrifuged at 3000 rpm for 10 min. Clear supernatant thus obtained was kept at − 80 °C for further study. The concentration of ascorbic acid was measured by UV spectrophotometer (UV-1201, Shimadzu, Kyoto, Japan) by using phenylhydrazine as an indicator by the previously mentioned method [32].

### Statistical analysis

The comparisons of variables between patient and control groups were performed by the independent sample t-test, the Mann-Whitney U test, and the Fisher’s exact test with a Bonferroni adjustment applied to the *p* value. Spearman’s correlation test was used to find out the correlation between the Ham-D score and non-enzymatic antioxidants and between the Ham-D score and amino acids. Cohen’s d effect sizes (d) were calculated for MDD patients versus HCs. Box-plot graphs were used to compare the study parameters between the cases and controls. SPSS version 23.0 (Armonk, NY: IBM Corp.) was used for all statistical analyses.

## Results

**Fig. 1:**
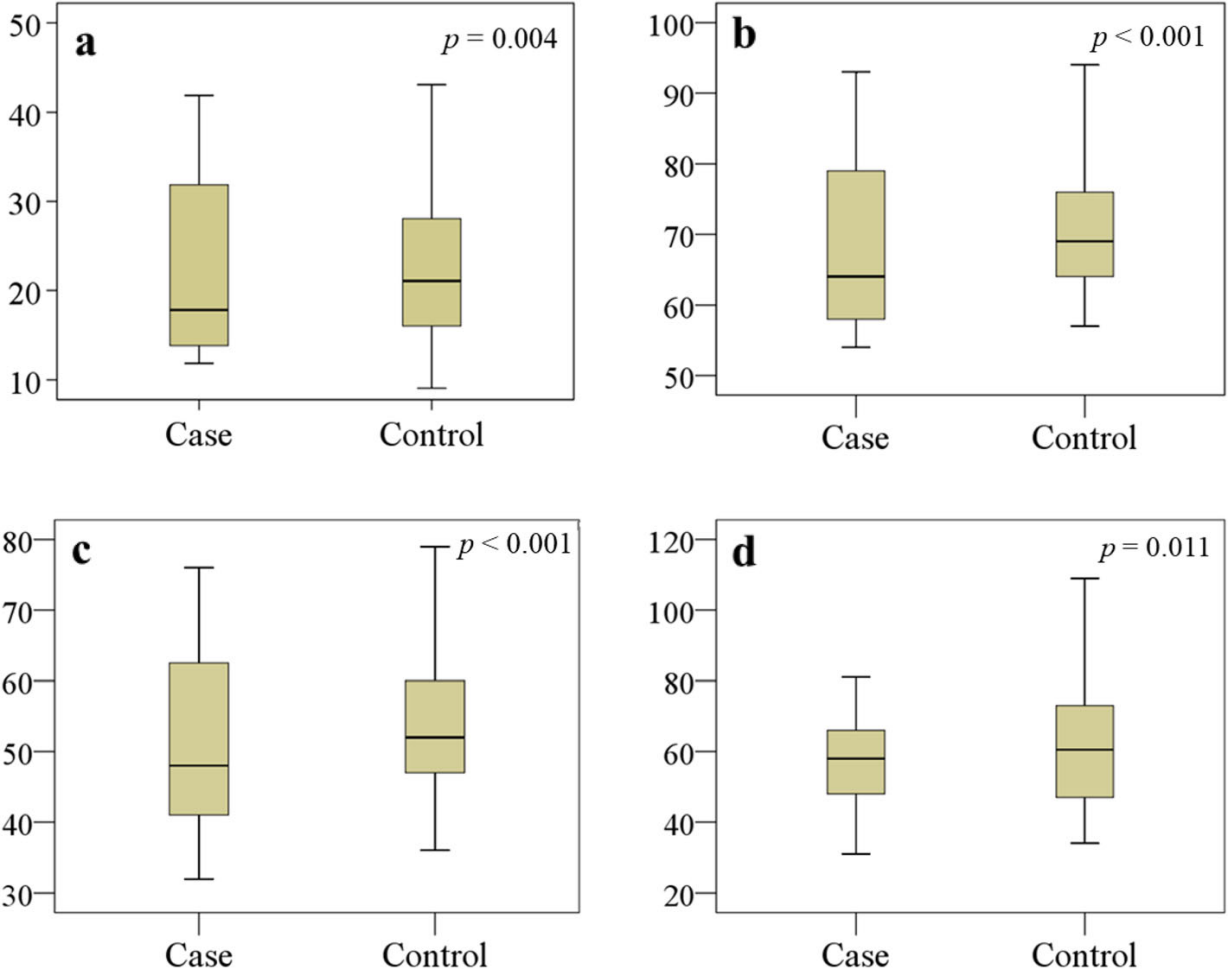
Changes in serum levels of amino acids (μmol/L) in the study population. Boxplot showing the median, maximum, and minimum value range. **a** Methionine, **b** Phenylalanine, **c** Tryptophan, **d** Tyrosine are lower in the patient group. Mann-Whitney U test was performed to present *p* values

**Fig. 2:**
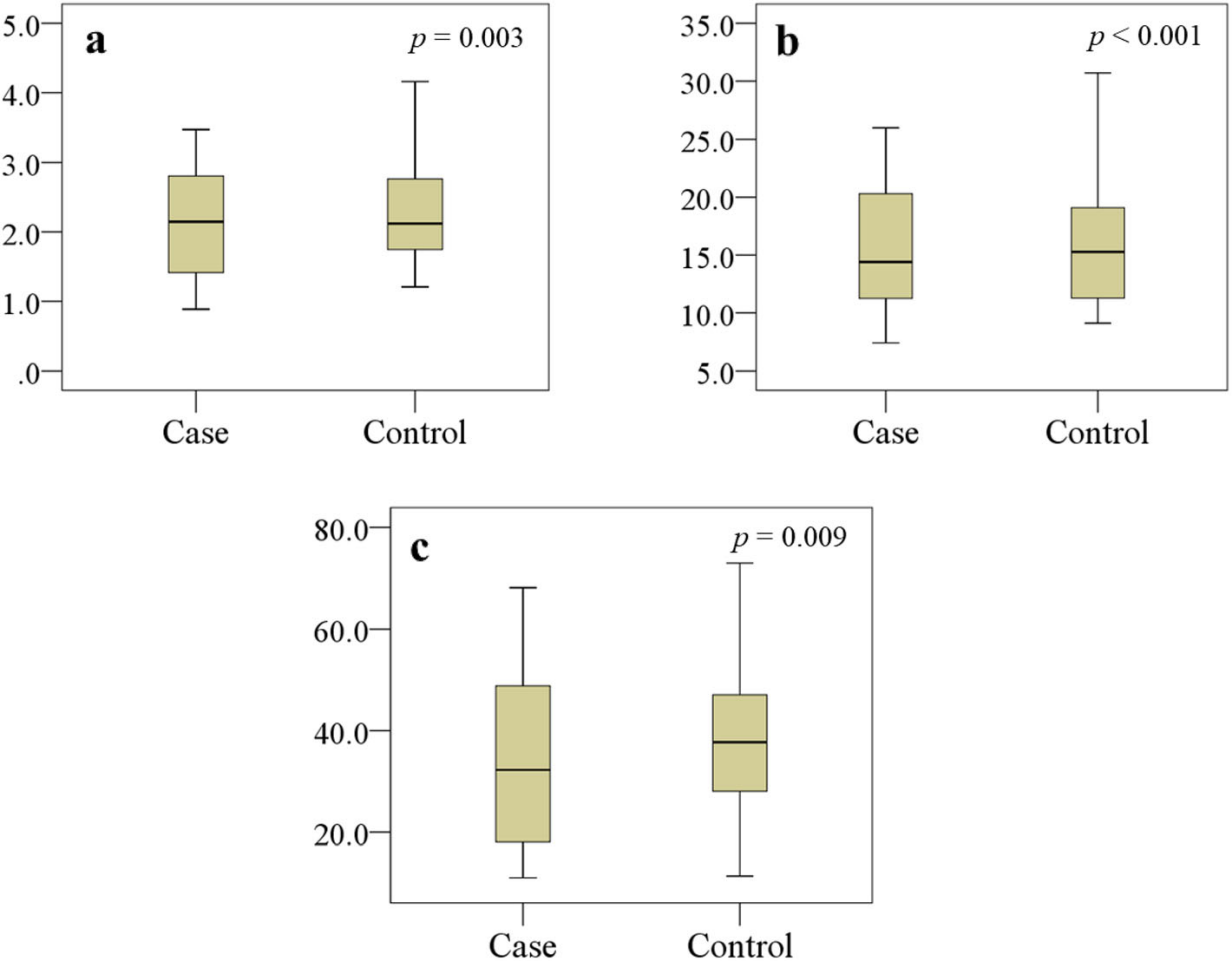
Variations in serum levels of antioxidant vitamins (μmol/L) in the study population. Boxplot showing the median, maximum, and minimum value range. **a** Vitamin A, **b** Vitamin E, **c** Vitamin C are lower in the patient group. Mann-Whitney U test was performed to present *p* values

Among the 302 randomly confirmed as MDD cases for this study, 31 patients were dropped due to the application of exclusion criteria, then 24 patients were not willing to participate in this study. Therefore, we recruited 247 MDD patients and 248 HCs. Socio-demographic and biographical characteristics are summarized in Table 1. No significant differences were observed between the groups by their age, sex, education, income, and BMI with effect size difference less than 0.20 in each case. Among all analyzed amino acids, serum levels of methionine, phenylalanine, tryptophan, and tyrosine were found notably declined in MDD patients as compared with HCs (*p* <  0.05) with Cohen’s d effect size differences of − 0.45, − 0.50, − 0.68, and − 0.21, respectively (Table 2). The distribution graphs of these amino acids were presented in Fig. 1. Our study found that serum concentrations of vitamin A, E and C were significantly lower in MDD patients in comparison to HCs (*p* <  0.05) with Cohen’s d effect size differences of − 0.27, − 0.65, and − 0.24, respectively (Table 3). The distribution graphs for antioxidant vitamins were presented in Fig. 2. Spearman’s correlation showed Ham-D scores were inversely related with serum levels of methionine (*r* = − 0.155, *p* = 0.015) and tyrosine (*r* = − 0.172, *p* = 0.007) in the patient group (Table 4). The Ham-D score showed no statistically significant positive or negative correlation with any of the analyzed amino acids or anti-oxidant vitamins in the patient group.

**Table 1:** The characteristics of the study population

Characteristics	MDD patients (*n* = 247)		Healthy controls (*n* = 248)		
	Median	Q_1_-Q_3_	Median	Q_1_-Q_3_	*p* value
Age in years	29.0	18.4–60.3	34.0	18.8–60.2	0.351^a^
Sex, male/female	91/156		102/146		0.407^b^
Education, illiterate/literate	32/215		26/222		0.721^b^
Monthly income (KBDT)	19.25	5.5–37.6	20.34	6.4–38.5	0.412^a^
BMI (kg/m^2^)	22.70	16.91–31.72	23.44	14.44–30.58	0.146^a^
Ham-D	17.4	8.43–27.72	3.64	2.17–6.53	< 0.001^a*^

^a^*p* values from independent sample t-test, ^b^*p* values from Fisher’s exact test, *p* is significant at the 0.05 level, ^*^ Bonferroni-corrected *p* value < 0.05: patients > controls (*p* < 0.001)

*KBDT* Kilo Bangladeshi taka, *BMI* Body mass index, Q_1_ lower quartile, Q_3_ upper quartile

**Table 2:** Serum concentrations of amino acids between healthy controls and MDD patients

Amino acids	MDD patients (*n* = 247)		Healthy controls (*n* = 248)		
(μmol/L)	Median	Q_1_-Q_3_	Median	Q_1_-Q_3_	*p* value
Alanine	233	202–492	238	203–502	0.065
Arginine	65.42	37.5–107	70.3	22.3–144	0.725
Aspartic acid	4.56	2.21–7.03	5.1	2.32–8.03	0.479
Cysteine	154	91.4–222	157	84.6–217	0.134
Glutamic acid	56.7	37.43–78.5	52.5	29.45–103	0.456
Glycine	205	114–476	210	104–464	0.107
Histidine	70.32	26.47–105	70.5	25.32-140	0.358
Isoleucine	51.3	41.94–80.7	53.6	37.43–99.4	0.212
Leucine	101	91.4–130	104	88.6–150	0.127
Lysine	159	92.1–305	157	90.3–290	0.837
Methionine	22.2	13.54–33.8	22.54	15.43–39.89	0.004^a^
Phenylalanine	64.8	54.41–93.73	69.7	53.58–115	< 0.001^b^
Proline	147	69.59–333	159	67.82–357	0.263
Serine	109	59.63–175	104	64.45–190	0.927
Threonine	128	62.52–210	136	65.73–206	0.088
Tryptophan	47.52	33.84–76.6	52.26	36.81–98.17	< 0.001^c^
Tyrosine	58.28	31.16–81.43	60.53	34.42–109	0.011^d^
Valine	196	156–311	189	149–309	0.752

*p* values from Mann-Whitney U test, *p* is significant at the 0.05 level, ^a^ Bonferroni-corrected *p* value < 0.05: patients < controls (*p* = 0.007), ^b^ Bonferroni-corrected *p* value < 0.05: patients < controls (*p* < 0.001), ^c^ Bonferroni-corrected *p* value < 0.05: patients < controls (*p* < 0.001), ^d^ Bonferroni-corrected *p* value < 0.05: patients < controls (*p* = 0.018)

Q1 lower quartile, Q3 upper quartile

**Table 3:** Serum concentrations of antioxidant vitamins between MDD patients and healthy controls

Antioxidant vitamins	MDD patients (*n* = 247)		Healthy controls (*n* = 248)		
(μmol/L)	Median	Q_1_-Q_3_	Median	Q_1_-Q_3_	*p* value
Vitamin A	2.23	1.36–3.29	2.51	1.70–3.65	0.003^a^
Vitamin E	15.56	9.11–31.50	18.00	11.00–25.60	< 0.001^b^
Vitamin C	30.28	11.04–68.10	38.75	11.39–72.96	0.009^c^

*p* values from Mann-Whitney U test, *p* is significant at the 0.05 level, ^a^ Bonferroni-corrected *p* value < 0.05: patients < controls (*p* = 0.005), ^b^ Bonferroni-corrected *p* value < 0.05: patients < controls (*p* < 0.001); ^c^ Bonferroni-corrected *p* value < 0.05: patients < controls (*p* = 0.010)

Q_1_ lower quartile, Q_3_ upper quartile

**Table 4:** Correlation study between altered research parameters and Ham-D scores in MDD patient

Correlation parameters	***r***	***p***
Methionine and Ham-D score	−0.155	0.015
Phenylalanine and Ham-D score	−0.120	0.060
Tryptophan and Ham-D score	− 0.122	0.056
Tyrosine and Ham-D score	−0.172	0.007
Vitamin A and Ham-D score	0.019	0.761
Vitamin E and Ham-D score	0.020	0.757
Vitamin C and Ham-D score	0.006	0.921

*r*, correlation coefficient; *p*, significance; Ham-D, Hamilton depression rating scale

*p* is significant at the 0.05 level; Negative values indicate the opposite correlation

## Discussion

The three substantial findings of these studies are 1. Reduced serum levels of four amino acids methionine, phenylalanine, tryptophan, and tyrosine in depression. 2. Decreased serum level of non-enzymatic antioxidants vitamin A, E, and C in depression. 3. An association of serum amino acids and antioxidants vitamin levels with the severity of depression.

Depression is thought to be linked with the scarcities of neurotransmitters such as serotonin and norepinephrine. Our brain synthesizes these neurotransmitters from certain amino acids. For instance, serotonin is synthesized from tryptophan, and norepinephrine is synthesized from tyrosine [33–36]. Low levels of serotonin and norepinephrine are directly involved in the pathogenesis of depression [37]. According to the monoamine hypothesis, a particular feature of depression develops due to the deficiency of corresponding neurotransmitters [38]. The decreased concentration of serum amino acid in MDD patients is due to the chronic catabolic status in the depression caused by the poor appetite which is a common symptom of MDD [39–42]. The lower levels of phenylalanine, tryptophan, and tyrosine explain that these amino acids are major contributors in the pathogenesis of depression which is similar to previous findings [14]. According to Xu et al., the plasma concentration of tryptophan is significantly decreased in MDD subjects [43, 44]. Another study shows that no relationship between tryptophan intake or tyrosine was established with depression [45, 46]. Although tryptophan, a precursor of serotonin can induce tranquillity and sleep by restoring serotonin levels [47–49]. A strong significant correlation between phenylalanine (*p* <  0.01) and depression was observed in patients with phenylketonuria [50]. Nonetheless, the number of past studies examining blood methionine levels in MDD is minor, and more studies are required [51]. The endogenous molecule SAMe is synthesized from methionine [52]. In comparison to neurological controls, it was found that cerebrospinal fluid SAMe levels were decreased in MDD [53]. De Berardis et al. have reported an advantageous effect of SAMe in MDD treatment [54].

Antioxidant vitamins play a vital role in the physiological process including neuroprotection, oxidative free radical production, and immune-modulatory functions. Neurodegeneration processes are associated with oxidative stress and many studies suggest that some neurological disorders due to oxidative stress may be prevented or cured by antioxidant vitamin therapy [55]. Vitamins C and E provide synergistic neuroprotection in the jejunum [56]. Our present study results about anti-oxidant vitamins are supported by several previous findings. Pandya et al. explained the potential cause of depression might be reduced level of antioxidants namely vitamin A, E, and C [25]. However, Maes et al. found depleted serum levels of vitamin E in depressed patients compared to healthy individuals [29]. Yet another report said the intensity of the disease is correlated with the decreased level of vitamin C [57]. Serum levels of total antioxidants were also reported to be significantly lowered in psychiatric patients than HCs [57, 58].

As interpreted above, reduced levels of some important amino acids and non-enzymatic antioxidant vitamins look like a reliable finding for MDD. By considering the earlier findings and accumulation of our results to them, we prudently suggest that these significant changes in serum level may contribute to the pathogenesis of major depression. The positive aspect of the present study is a large and homogeneous study population.

Though the evaluated parameters in MDD patients compared to HCs furnished in the field of biological psychiatry, the present study had few limitations. We did not investigate the impact of dietary supplementation and lifestyle on the analyzed parameters. This study enrolled all the patients from a single institution that may not represent the national picture. The serum amino acid and vitamin levels were measured only once before treatment. Therefore, to measure changes of these parameters in serum levels after treatment with antidepressants was not possible. Thus, this study should be treated as preliminary and further studies with more homogenous samples from different geographical locations are required to support our findings. Despite these limitations, we hope our study findings will aid the recently available methods for the proper diagnosis and management of depression.

## Conclusion

To the best of our knowledge, this is the first-ever study on Bangladeshi patients to figure out the association of serum levels of amino acids and depleted non-enzymatic antioxidants with depression. Reduced serum levels of few amino acids and non-enzymatic antioxidants were observed in major depression and this might happen due to abnormal neurological physiology. These changes in serum levels arise independently and may be associated with the risk for developing MDD.

## Data Availability

Data supporting our findings are available from the corresponding author on reasonable request.

## Abbreviations

ATP: Adenosine triphosphate
BMI: Body mass index
BSMMU: Bangabandhu Sheikh Mujib medical university
CRH: Corticotropin-releasing hormone
DSM-5: Diagnostic and statistical manual of mental disorders, 5th edition
Ham-D: Hamilton depression rating scale
HCs: Healthy controls
MDD: Major depressive disorder
RP-HPLC: Reverse-phase high-performance liquid chromatography
SAMe: S-adenosyl-l-methionine
SPSS: Statistical package for the social sciences
TCA: Trichloroacetic acid
UV: Ultraviolet
